# Editorial: Interactions between tissues and kingdoms and interplay with environmental factors: impact on metabolic health and diseases

**DOI:** 10.3389/fendo.2025.1665747

**Published:** 2025-08-12

**Authors:** Giselle Adriana Abruzzese, Victòria Ceperuelo-Mallafré

**Affiliations:** ^1^ Liver Disease Lab, Center for Cooperative Research in Biosciences (CIC bioGUNE), Basque Research and Technology Alliance (BRTA), Derio, Bizkaia, Spain; ^2^ Departament de Medicina i Cirurgia, Universitat Rovira i Virgili (URV), Reus, Spain; ^3^ Hospital Universitari Joan XXIII de Tarragona, Institut d’Investigació Sanitària Pere Virgili (IISPV)-CERCA, Tarragona, Spain; ^4^ CIBER de Diabetes y Enfermedades Metabólicas Asociadas (CIBERDEM)-Instituto de Salud Carlos III (ISCIII), Madrid, Spain

**Keywords:** metabolic diseases, endocrine disorders, inter-kingdom communication, tissues and organs crosstalk, metabolism

Communication between tissues via secreted factors is essential for coordinating whole-body metabolism. These include peptides, lipids, small molecules, and other factors such as extracellular vesicles capable of transmitting information about the metabolic state between distant organs and tissues. The dysregulation of one or more of these signaling axes lies at the basis of the development of metabolic diseases. Understanding how organs communicate through secreted factors is crucial for discovering new and promising therapeutic targets.

This Research Topic gathers contributions that highlight the role of major players in tissue crosstalk and metabolic health regulation, emphasizing organ and system interactions as a promising approach for the management of metabolic diseases.

In recent years, advances in integrative medicine have led to new insights into the contributions of some tissues and their derived molecules to physiology and metabolic diseases. Such is the case of adipose tissue. Adipose tissue, apart from storing energy, has been recognized as a complex and dynamic endocrine organ that regulates energy homeostasis and other important physiological processes. Extracellular vesicles (EVs) derived from adipose tissue represent an essential part of the adipose secretome, participating in autocrine and paracrine interactions within the different cells in it as well as in endocrine communications. The review by Wang et al. provides a comprehensive evaluation of the content and function of different exosomes (a subtype of EVs) within adipose tissue, highlighting the complex regulatory effects. The authors emphasize the diverse roles of these exosomes in modulating inflammation, adipogenesis, tumor progression, and insulin resistance. Moreover, they influence both physiological and pathological processes in distant organs such as the muscles, skin, hypothalamus, and kidneys. Interestingly, the cargo of these exosomes differs between healthy, obese, and diabetic individuals, suggesting their potential as diagnostic biomarkers and therapeutic targets. However, clinical translation remains limited by several challenges, including difficulties in standardizing exosome isolation and storage, variability in therapeutic efficacy based on donor condition, and concerns about potential immunogenicity or adverse effects. Therefore, despite their therapeutic potential, the clinical application of adipose tissue-derived exosomes will require robust protocols for quality control, deeper mechanistic understanding, and extensive safety validation.

Increasing recognition is being given to environmental factors and health habits, such as sleep, diet, and physical activity, as contributors to metabolic homeostasis and health. Wang et al., for example, explore the role of sleep as a central regulator of glucose metabolism. The authors investigated the potential benefits of sleep compensation, through weekend catch-up sleep, in individuals with diabetes, showing that short sleep compensation can improve glucose metabolic dysregulation in diabetic individuals. The study drew attention to an important but still understudied field, highlighting sleep as a potential key modulator in metabolic functions. This study gains relevance in a context in which sleep loss or disruption is being recognized as a metabolic disorder (1) by affecting the neuroendocrine system and negatively impacting energy balance. Further research is needed to deepen the understanding of the mechanisms related to the role and control of sleep in metabolic health and diseases, especially in the context of energy impairment diseases such as obesity and diabetes.

A key contributor to metabolic health and disease is the gut microbiome, which further modulates inter-organ communication, not only in the liver-gut axis but also by affecting other distant organs and influencing a wide range of physiological functions (2). A growing body of evidence positions the gut microbiota as a dynamic endocrine organ, highlighting its bidirectional communication with host cells and its essential role in metabolic regulation via hormone modulation, metabolite synthesis, and immune signaling. Despite this growing recognition, critical questions remain regarding the specific microbial taxa involved, their functions, and their interaction with host pathways. Xue et al. explored the effects of orlistat, a weight-loss drug, on gut microbial communities in a mouse model of diet-induced obesity. Their findings reveal that orlistat reshapes microbial diversity and composition, correlating with improved metabolic markers like fasting glucose and gut hormones GLP-1 and GIP. These results suggest that part of orlistat’s efficacy may be mediated via microbiota modulation. However, further mechanistic studies are needed to confirm causal links beyond correlative analyses.

In addition to modulating digestive hormones, the gut microbiome also influences sex hormone regulation. Wang et al. review how the gut microbiome impacts estrogen regulation in postmenopausal women. They highlight that decreased microbial diversity after menopause may reduce the activity of β-glucuronidase, disrupting estrogen reabsorption through enterohepatic circulation. This alteration is associated with metabolic, cognitive, and skeletal disorders commonly observed during this period. Furthermore, Mei et al. highlight the potential role of the microbiota in polycystic ovary syndrome (PCOS). PCOS, a common endocrine disorder characterized by hyperandrogenism, affects women of reproductive age and is associated with high androgen levels and disrupted gonadotropin regulation (3). The authors discuss how microbiota diversity and its derived metabolites may contribute to insulin resistance, a key metabolic feature of PCOS, based on evidence from both animal models and clinical studies.

Expanding on the interplay between the gut microbiota, hormones, and metabolism, Chen et al. review the use of probiotics in addressing obesity during adolescence, a critical period marked by hormonal changes. They point out the limitations of mouse models for studying puberty-related obesity and provide clinical data showing that probiotic treatments may improve metabolic parameters. However, the authors emphasize the need for further research, particularly in diverse populations, given the variability in outcomes. To better implement probiotics and prebiotics in the clinical management of metabolic diseases, large-scale, multicenter studies are needed to assess long-term effects and define optimal treatment conditions.

Together, these contributions underscore the vital role of inter-tissue and inter-kingdom communication in metabolic regulation. Although they do not comprehensively address the full complexity of these interactions, they highlight the need for further exploration of inter-tissue and inter-kingdom signaling. Deeper mechanistic insights into the host–microbe and other key pathways are essential and could ultimately pave the way for more precise and personalized therapies for complex metabolic disorders.
